# The Accuracy of Wrist-Worn Photoplethysmogram–Measured Heart and Respiratory Rates in Abdominal Surgery Patients: Observational Prospective Clinical Validation Study

**DOI:** 10.2196/40474

**Published:** 2023-02-20

**Authors:** Jonna A van der Stam, Eveline H J Mestrom, Jai Scheerhoorn, Fleur E N B Jacobs, Simon Nienhuijs, Arjen-Kars Boer, Natal A W van Riel, Helma M de Morree, Alberto G Bonomi, Volkher Scharnhorst, R Arthur Bouwman

**Affiliations:** 1 Department of Biomedical Engineering Eindhoven University of Technology Eindhoven Netherlands; 2 Clinical Laboratory Catharina Hospital Eindhoven Netherlands; 3 Expert Center Clinical Chemistry Eindhoven Eindhoven Netherlands; 4 Department of Anesthesiology Intensive Care & Pain Medicine Catharina Hospital Eindhoven Netherlands; 5 Department of Surgery Catharina Hospital Eindhoven Netherlands; 6 Department of Medical Physics Catharina Hospital Eindhoven Netherlands; 7 Department of Vascular Medicine Amsterdam University Medical Centers Amsterdam Netherlands; 8 Patient Care & Monitoring Department Philips Research Eindhoven Netherlands; 9 Department of Electrical Engineering Eindhoven University of Technology Eindhoven Netherlands

**Keywords:** Telemetry, monitoring, photoplethysmography, PPG, photoplethysmogram, wearable monitoring, vital parameter, wearable sensor, sensor, heart rate, respiratory Rate, respiration, respiratory, breathing, monitoring, wearable, postoperative, post-operative, vital sign

## Abstract

**Background:**

Postoperative deterioration is often preceded by abnormal vital parameters. Therefore, vital parameters of postoperative patients are routinely measured by nursing staff. Wrist-worn sensors could potentially provide an alternative tool for the measurement of vital parameters in low-acuity settings. These devices would allow more frequent or even continuous measurements of vital parameters without relying on time-consuming manual measurements, provided their accuracy in this clinical population is established.

**Objective:**

This study aimed to assess the accuracy of heart rate (HR) and respiratory rate (RR) measures obtained via a wearable photoplethysmography (PPG) wristband in a cohort of postoperative patients.

**Methods:**

The accuracy of the wrist-worn PPG sensor was assessed in 62 post–abdominal surgery patients (mean age 55, SD 15 years; median BMI 34, IQR 25-40 kg/m^2^). The wearable obtained HR and RR measurements were compared to those of the reference monitor in the postanesthesia or intensive care unit. Bland-Altman and Clarke error grid analyses were performed to determine agreement and clinical accuracy.

**Results:**

Data were collected for a median of 1.2 hours per patient. With a coverage of 94% for HR and 34% for RR, the device was able to provide accurate measurements for the large majority of the measurements as 98% and 93% of the measurements were within 5 bpm or 3 rpm of the reference signal. Additionally, 100% of the HR and 98% of the RR measurements were clinically acceptable on Clarke error grid analysis.

**Conclusions:**

The wrist-worn PPG device is able to provide measurements of HR and RR that can be seen as sufficiently accurate for clinical applications. Considering the coverage, the device was able to continuously monitor HR and report RR when measurements of sufficient quality were obtained.

**Trial Registration:**

ClinicalTrials.gov NCT03923127; https://www.clinicaltrials.gov/ct2/show/NCT03923127

## Introduction

Alterations in vital parameters can often be found hours before a life-threatening event occurs [1-7]. In current clinical practice, postoperative monitoring often consists of a period of continuous monitoring in an intensive care or postanesthesia care unit, followed by an admission to a general ward. Since continuous monitoring of vital parameters is not present in the general ward, nursing staff performs the so-called spot checks to monitor the patient’s vital parameters. During these spot checks, the nursing staff measures several vital parameters, often followed by manual entry or calculation of an early warning score such as the Modified Early Warning Score, to identify patients at risk of deterioration [8]. In clinical practice, these spot checks form a considerable workload, and vital parameters, especially respiratory rate (RR), are often poorly registered [9,10]. Additionally, as the name implies, these spot checks capture only vital parameters at a specific moment in time, and vital parameters during the rest of the day remain unknown. Alternatively, wearable sensors could be used to unobtrusively and continuously measure vital parameters in postoperative patients. However, their accuracy in postoperative patients should be established prior to introduction in clinical practice.

One type of wearable sensor that can monitor a patient’s vital parameters is a photoplethysmography (PPG) wristband. This type of sensor measures the intensity of the light reflected from the skin, which indicates changes in the blood volume in peripheral circulation, to determine both heart rate (HR) and RR [11]. Wrist-worn PPG sensors have potential for use as a continuous, unobtrusive monitoring system in low-acuity settings such as the general ward.

A few studies have reported the accuracy of other PPG-based wearables in hospitalized patients; however, these trials only studied the measurement of HR [12-15]. Additionally, the accuracy of wrist plethysmography devices for HR measurements in a perioperative cohort was previously investigated and found to be clinically acceptable [15]. However, as both HR and RR have been identified as important parameters for the prediction of clinical deterioration, accuracy for both vital parameters should be established [16]. Therefore, this study aims to assess the accuracy of a wrist-worn PPG device for measuring both RR and HR in postoperative patients.

## Methods

### Study Population

These analyses were performed with a subpopulation of Transitional Care Study 3 (TRICA; ClinicalTrials.gov NCT03923127)—a single-center study on wearable monitoring in postoperative patients in a tertiary hospital [17,18]. All adult patients scheduled for major abdominal oncological or bariatric surgery from April 2019 to August 2020 who were willing and able to sign informed consent were eligible for participation. Patients who met any of the following criteria were not included: being pregnant or breastfeeding, having an allergy to tissue adhesives, having an antibiotic-resistant skin infection, having an active implantable device, or having any skin condition at the area of application of the devices. This subanalysis describes 68 postoperative patients, and inclusion into this subanalysis for accuracy of the wearable sensor was based on the availability of research personnel and real-time data logging equipment.

### Ethics Approval

The trial was approved by the medical ethical committee METC Máxima MC, Veldhoven, The Netherlands (W19.001).

### Data Collection

The wearable PPG wristband device, ELAN, was equipped with a Philips Cardio and Motion Monitoring Module (CM3, Philips Electronic Nederland BV), which contains a PPG and 3-axial accelerometer sensor. The PPG sensor measures the intensity of the green light scatter-reflected from the skin to determine changes in blood volume in the peripheral circulation with a sampling frequency of 32 Hz [19]. From the obtained PPG signal, HR and RR were determined using previously published algorithms, the RR measurements are derived from interbeat interval variability and PPG amplitude [20]. Additionally, the device reports a quality index with each measured vital value, which mostly captures the signal-to-noise ratio [15]. Only vitals with a quality index of 4 (range 0-4), are considered to be of high quality and can be included in further analysis.

Shortly after surgery, the PPG wristband was applied to the patient’s wrist in the postanesthesia care unit (PACU) or intensive care unit (ICU), depending on where the patient was recovering immediately after surgery. The wristband then continuously collected both HR and RR.

As a ground truth, the electrocardiogram (ECG)-based HR and capnography-based RR signals of 68 patients were extracted from the bedside monitor in the PACU or ICU. These signals were saved in real time for offline processing, allowing comparison between the HR and RR measured by the PPG wristband and the reference monitor. In the PACU, vital parameters from the CAR-ESCAPE monitor B650 (GE Healthcare) were extracted using iCollect software (GE Healthcare) with a sampling frequency of 250 Hz for ECG and 1 Hz for RR. In the ICU, vital parameters were extracted from the Philips IntelliVue MP70 monitor using IntelliVue software (Philips) with a sampling frequency of 100 Hz for ECG and 0.1 Hz for RR. HR was derived from the ECG on second-to-second bases using QRS detection algorithms, RR was obtained using the patient monitors’ algorithms.

### Data Analysis

The obtained vital parameters from the PPG wristband and the reference monitors were synchronized using a means of cross-correlation on the HR signals, and synchronized signals were visually inspected and corrected if necessary. Patients with a reference recording length shorter than 15 minutes were excluded from the analysis.

Low-quality measurements were excluded from both the PPG and monitor data. For the PPG wristband vitals, a low quality index can originate from motion artefacts or a low signal-to-noise ratio. For HR and RR, detection of arrhythmia using an arrhythmia detection algorithm would also lead to a low quality score [21]. For the reference monitor, the logged ECG and capnography signals were visually inspected to identify low-quality measurements, based on assessment of the temporal sequence.

Baseline characteristics are expressed as mean (SD) or, in case of nonnormally distributed values, as median (IQR) values. Agreement between the PPG wristband and reference monitor measurements on a second-to-second basis was visualized using Bland-Altman plots [22]. As multiple observations from the same patients were analyzed, the bias and limits of agreement were calculated using the method for repeated measures of Zou et al [23]. Additionally, the 95% CIs around the limits of agreement were assessed using MOVER [23].

According to the American National Standards Institute consensus standard, the error for HR measurements should be ≤10% or ≤5 bpm. In this analysis, an error of ≤5 bpm for HR and ≤3 rpm for RR was considered clinically acceptable. Additionally, Clarke error grid analysis was performed to quantify the implications of the difference between the vitals measured by the reference monitor and the PPG wristband. Clarke error grid analysis was originally developed for blood glucose measurements, and the boundaries of the different zones were adapted on the basis of the Modified Early Warning Score protocol used in our hospital [8,17,24,25].

## Results

**Figure 1:**
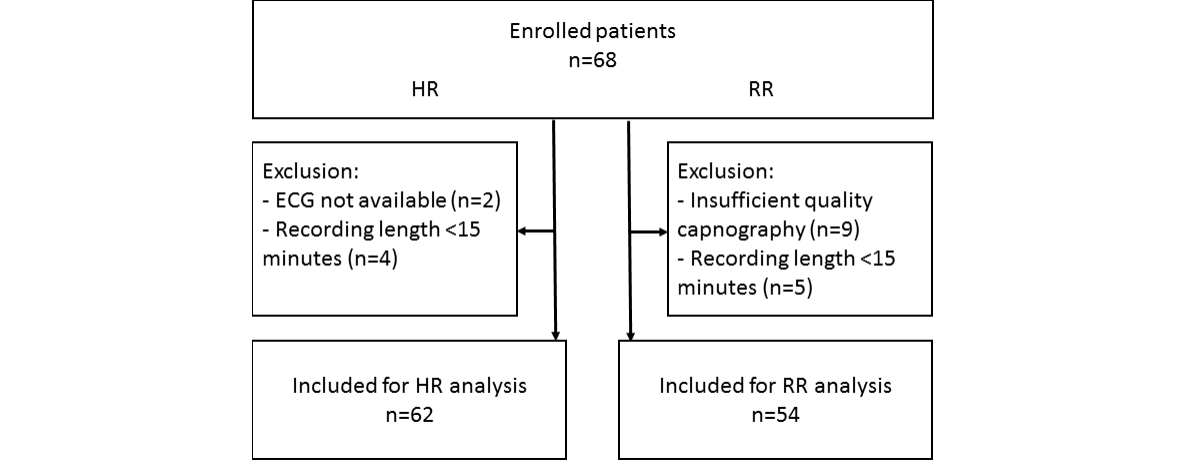
Flowchart of patient inclusion for heart rate (HR) and respiratory rate (RR) analysis. A total of 62 patients were included in the data analysis, of whom 8 were only included in the HR analysis and 54 were included in both analyses. ECG: electrocardiography.

In total, 68 postsurgical patients were enrolled, of whom 6 were excluded from HR analysis due to either unavailable ECG reference (n=2) or a recording length of less than 15 minutes (n=4). For RR analysis, 14 patients were excluded from further analysis due to either lack of sufficient quality capnography reference data (n=9) or insufficient recording length (n=5) (Figure 1). The characteristics of the included population are shown in (Table 1).

**Table 1:** Population demographics (N=62).

Variable			Value
Female, n (%)			33 (53)
Age (Years), mean (SD)			55 (15)
BMI (kg/m^2^), median (IQR)			34 (25-40)
**Surgery type, n (%)**			
	Gastric bypass	21 (34)	
	Gastric sleeve	9 (15)	
	Esophagectomy	7 (11)	
	Hyperthermic intraperitoneal chemotherapy	9 (15)	
	Pancreatectomy	4 (6)	
	Low anterior resection or abdominoperineal resection with intraoperative radiation therapy	6 (10)	
	Low anterior resection or abdominoperineal resection without intraoperative radiation therapy	4 (6)	
	Debulking	2 (3)	
Duration of surgery (minutes), median (IQR)			144 (76-342)
Postoperative admission to the intensive care unit, n (%)			27 (43)

### HR Assessment

**Figure 2:**
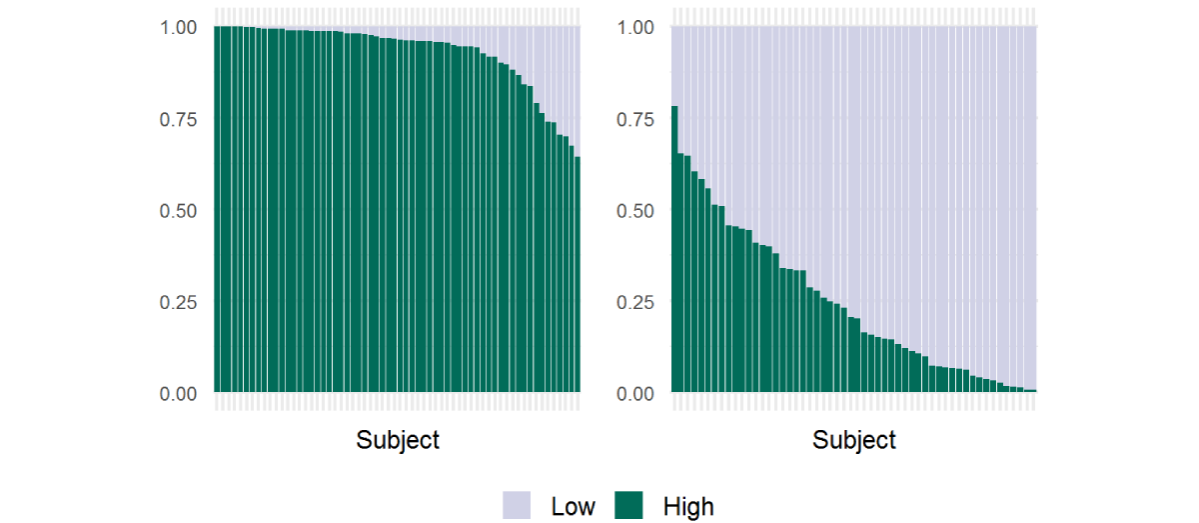
Availability of photoplethysmography wristband data of high-quality data for heart rate (left) and respiratory rate (right) expressed as the percentage of seconds with high- and low-quality data.

For HR assessment, a total of 146 hours of data, from both the PACU or ICU patient monitor and the wearable sensor, were collected in 62 patients. Per patient, a median of 1.2 hours of data (range 16 minutes to 10 hours) were collected. Overall, 492,987 (94%) of the PPG wristband data points were of sufficient quality to be included in the analysis. As shown in (Figure 2), the percentage of sufficient-quality HR data per patient varied among patients, and a median of 96% (IQR 92%-99%) of high-quality HR data were obtained. The gaps without high-quality HR data ranged from a length of 1 second to 7.2 minutes, and 96% of the gaps were of <60 seconds.

**Figure 3:**
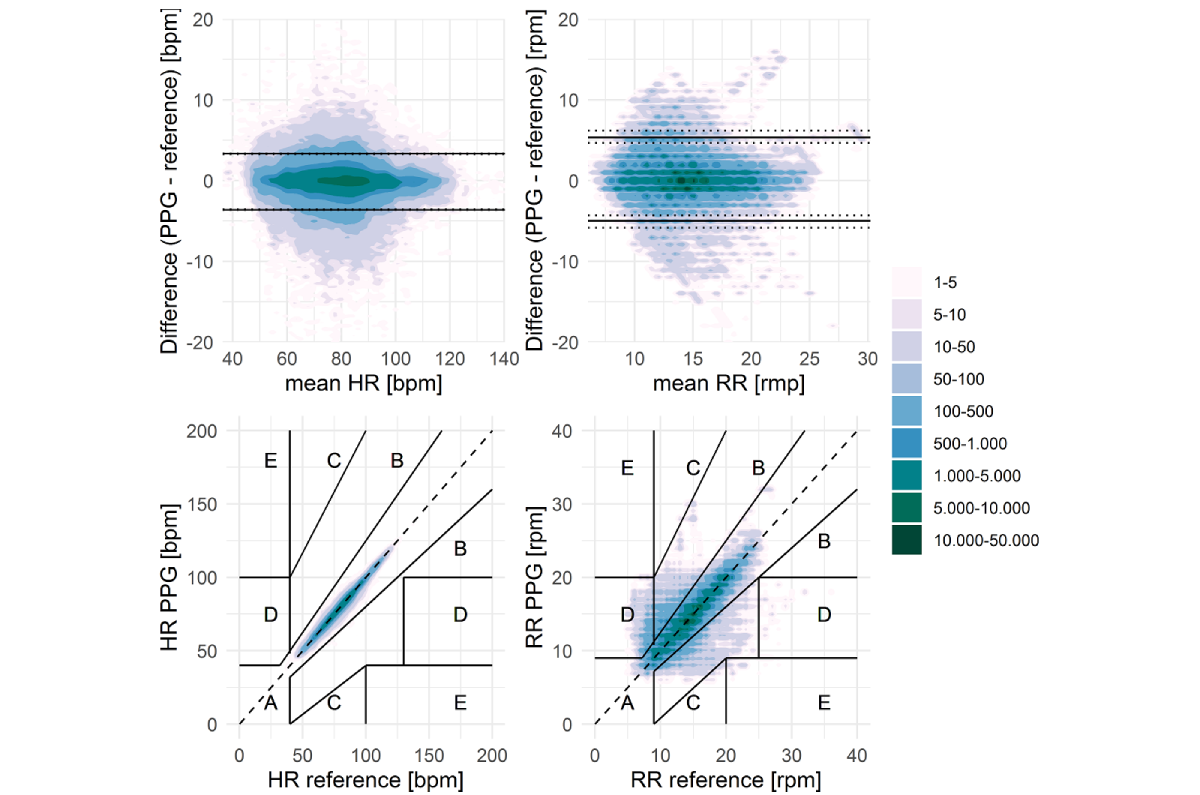
Bland-Altman (top) and Clarke error grid (bottom) plots of the vital parameters obtained from the photoplethysmography (PPG) wristband and reference monitor, each data point represents 1 second. The upper figures depict Bland-Altman analysis for heart rate (HR; left) and respiratory rate (RR; right). Limits of agreement are indicated by the black lines, dashed lines represent the 95% CIs of the limits of agreement. The bottom figures depict the Clarke error grid analysis for HR (left) and RR (right) comparing the measurements of the reference monitor (x-axis) and the PPG wristband (y-axis). Zone A represents data points that differ less than 20% from the reference or are correctly identified as bradycardia or bradypnea. Zone B represents data points that differ by more than 20% but would not cause unnecessary treatment. Zone C represents points that would lead to unnecessary treatment for patients with normal vital parameters. Zone D represents failure to detect bradycardia or bradypnea, or tachycardia or tachypnea. Zone E represents data points where bradycardia or bradypnea and tachycardia or tachypnea are confused.

Bland-Altman and Clarke error grid analysis were used to assess the accuracy of the PPG wristband-measured HR (Figure 3 and Table 2). Bland-Altman analysis showed a bias of –0.15 bpm and limits of agreement of –3.62 to 3.32 bpm. As the limits of agreement lie within the predefined ≤5 bpm, the PPG wristband HR measurements met the required accuracy. Clarke error grid analysis showed that 100% (484,085 data points) of the measurements were within the clinically acceptable zones A and B, indicating that no incorrect treatment would result from the use of PPG wristband–derived HR values. Splitting the data on the basis of the unit patients were admitted to (ICU vs PACU) showed comparable availability of good-quality PPG measurements and similar bias and limits of agreement (Multimedia Appendix 1).

**Table 2:** Agreement and clinical accuracy of heart rate (HR) and respiratory rate (RR) measured by the photoplethysmography (PPG) wristband compared to those of the reference monitor.

			HR		RR
**Data availability**					
	Patients, n	62		54	
	Measurements, n	526,833		495,217	
	Good-quality PPG wristband, n (%)	492,987 (94)		170,383 (34)	
	Good-quality reference, n (%)	515,991 (98)		367,092 (74)	
	Both good quality, n (%)	484,096 (92)		128,816 (26)	
**Bland-Altman analysis**					
	Pearson *r*	0.99		0.80	
	Bias (bpm/rpm), mean (SD)	–0.15 (1.8)		0.17 (2.6)	
	Lower limit of agreement (bpm/rpm), lower limit (95% CI)	–3.62 (–3.7 to –3.6)		–4.99 (–5.8 to –4.3)	
	Upper limit of agreement (bpm/rpm), upper limit (95% CI)	3.32 (3.3 to 3.4)		5.33 (4.7 to 6.2)	
	Within 5 bpm or 3 rpm, %	98		93	
**Clarke error grid analysis, n (%)**					
	A	483,716 (99.9)		115,434 (89.6)	
	B	369 (0.1)		10,781 (8.4)	
	C	0 (0)		61 (0)	
	D	11 (0)		2499 (1.9)	
	E	0 (0)		41 (0)	
	A+B	484,085 (100)		126,215 (98.0)	

### RR Assessment

For RR, a total of 138 hours of data, from both the PACU or ICU patient monitor and the wearable sensor, were collected from among 54 patients. A median of 1.2 hours (range 16 minutes to 11 hours) of data were collected per patient. Overall, 170,383 (34%) of the PPG wristband RR measurements were of sufficient quality to be included in further analysis. Figure 2 shows the availability of high-quality data per patient, a median of 20% (IQR 7%-40%) of sufficient-quality RR data were obtained. The gaps without high-quality RR data ranged from a length of 1 second to 67 minutes, and 81% of the gaps were of <60 seconds.

Bland-Altman analysis of the PPG wristband–measured RR showed a bias of 0.17 rpm and limits of agreement of –4.99 to 5.33 rpm. As 93% of the RR measurements met the predefined ≤3 rpm, the limits of agreement were wider than the predefined ±3 rpm. Clarke error grid analysis showed that 98% of the data points were within the clinically acceptable zones A and B, indicating that the differences between the PPG wristband RR and reference monitor only have limited clinical implications. Most of the remaining 2% of data points lie within zone D, which indicates failure to detect impaired RR either due to failure to detect bradypnea (1.85%) or tachypnea (0.09%). Splitting the data based on the unit patients were admitted to (ICU vs PACU) showed a numerically higher percentage of available good-quality PPG measurements in the ICU and numerically wider limits of agreement in the PACU (Multimedia Appendix 1).

## Discussion

### Principal Findings

The use of wearable sensors to monitor hospitalized patients is rapidly attracting attention in the clinical community. However, prior to the introduction of these devices in clinical practice, their performance in the patient population of interest needs to be established. As postoperative patients are currently only monitored using spot checks for the duration for which they are in the general ward, this population could benefit from wearable monitoring. This study focused on the performance of a wearable PPG wristband for the measurement of HR and RR in postoperative patients.

For HR, the device was able to accurately measure the vital parameter as the bias and limits of agreement were within the predefined ≤5 bpm. Any differences between the PPG wristband and reference monitor were found to be clinically acceptable since 100% of the measurements were within zones A and B of the Clarke error grid. Additionally, the wearable PPG sensor would be feasible in terms of data availability for HR as the device only reported low quality for 4% of the HR measurements.

For RR, 93% of the included measurements were within the predefined ≤3 rpm and while the bias was within this threshold, the limits of agreement were wider than the predefined cutoff. However, as 98% of the included measurements lie within zones A and B of the Clarke error grid, the device does provide clinically acceptable measurements for the large majority of the included measurements. The detection of RR by the wrist-worn PPG is easily corrupted by motion artifacts, leading to the exclusion of 66% of the measurements due to a low quality index. Therefore, the wearable PPG wristband would be unable to continuously measure RR. However, with 81% of the gaps of <1 minute, the device is able to measure RR more frequently than the current intermittent monitoring and therefore could potentially replace the RR measurements during the spot checks that are currently performed manually 3 times a day.

### Comparisons to Prior Work

The accuracy of HR measurements by the same PPG wristband was previously studied in the PACU of our hospital. In the cohort of this study, the clinical accuracy in the PACU and ICU was confirmed with comparable results [15]. The accuracy of another wrist-worn PPG personal fitness tracker sensor for the monitoring of HR in hospitalized patients was previously studied by Kroll et al [12], who reported a bias of –4.7 and lower and upper limits of agreement of –31 and 21, respectively. Additionally, 73% of their measurements met the desired ≤5 bpm. Our findings with the ELAN PPG wristband show better agreement with the reference signal than their findings using the Fitbit Charge HR. The accuracy of another wrist-worn PPG sensor, the CardiacSense, in ambulatory patients was studied by Hochstadt et al [13]. As they reported their findings regarding the length of peak intervals rather than HR, comparison of results is difficult.

Limited data on the accuracy of RR measurement using PPG in clinical settings are available. Touw et al [26] studied the accuracy of finger-cuff PPG RR measurements in patients receiving procedural sedation and analgesia and found a bias of –2.0 rpm with limits of agreement from –12.4 to 8.4 rpm. Compared to their findings, the PPG wristband used in this study can measure RR with a smaller bias and smaller limits of agreement. Haveman et al [27] compared upper arm–worn wearable PPG measurements of HR and RR to manual those performed by nursing staff. They found a moderate relationship for HR and a poor relationship for RR. However, their results cannot be easily compared to ours as gold-standard measurements were unavailable in their cohort. Additionally, Haveman et al [28] described lower accuracy and data availability for upper arm–measured PPG RR during activity in volunteers. Patient activity level could therefore be a potential factor that relates to the differences between the RR in the ICU and the PACU. However, as the postoperative unit a patient is admitted to is chosen on the basis of surgery type, severity, and patient characteristics, this trial does not allow drawing conclusions regarding the origin of these differences. Papini et al [29] studied respiratory activity in a sleep-disordered population using a wrist-worn PPG device. They found a median correlation of 0.62 and a median per patient coverage of 75.3%. Comparison of the accuracy to our findings is complicated as we reported a correlation over the entire data set; however, an overall correlation of 0.80 in this study indicates a better agreement between the 2 RR measurements. However, their median per-patient coverage of 75.3% clearly outperforms the 20% found in the present population.

### Strengths and Limitations

This analysis was performed in a real-world, clinically relevant patient population, as postoperative patients could benefit from wearable monitoring in low-acuity care settings such as the surgical ward. However, this study had some limitations. First, while capnography is the gold standard for RR monitoring, a good-quality reference RR signal could not be obtained for 9 patients, and for the patients who could be included, 26% of the capnography data had insufficient quality to be included. Second, the data for this trial were collected in the PACU and ICU rather than the general ward. However, we believe that our findings could reasonably be transferred to the general ward as patients became alert and mobile during their stay in these recovery units. Third, the analysis of trending ability of the device could be an interesting addition to the data analysis and can be included in future research if longer monitoring times of both the wearable and reference monitor are available.

### Future Directions

Other potential future clinical applications of PPG wearables include the measurement of activity level, blood pressure, HR variability, energy expenditure, and the detection of atrial fibrillation [21,30-33]. In future clinical use, PPG wristbands thus have the potential to provide information on even more aspects of the patients’ health status. This study shows that the ELAN PPG wristband can continuously measure HR with clinically acceptable accuracy. For RR, the device can perform clinically accurate measurements, but, due to limited coverage, can only be used to perform intermittent measurements.

### Conclusions

The wearable PPG wristband can measure HR accurately and with sufficient coverage in postoperative patients. For RR, the large majority of the included data were clinically acceptable; however, the coverage of sufficient-quality RR measurements was low. Therefore, the PPG wristband would be able to perform continuous monitoring of HR and also report RR when sufficient-quality measurements are obtained. Before implementing such PPG-based wearable devices in clinical practice, both accuracy and coverage should be considered.

## Appendix

Multimedia Appendix 1 Results split based on unit the patients were admitted to postoperatively.

## Abbreviations

ECG: electrocardiography
HR: heart rate
ICU: intensive care unit
PACU: postanesthesia care unit
PPG: photoplethysmography
RR: respiratory rate
