# Supplementary material for: The Accuracy of Wrist-Worn Photoplethysmogram–Measured Heart and Respiratory Rates in Abdominal Surgery Patients: Observational Prospective Clinical Validation Study
Source: JMIR Perioper Med. 2023 Feb 20;6:e40474. doi: 10.2196/40474 (PMC9989911; doi:10.2196/40474)
Supplement: Multimedia Appendix 1 [file periop_v6i1e40474_app1.pdf]

Supplemental materials

Results split based on unit the patients were admitted to postoperatively.

Intensive Care Unit

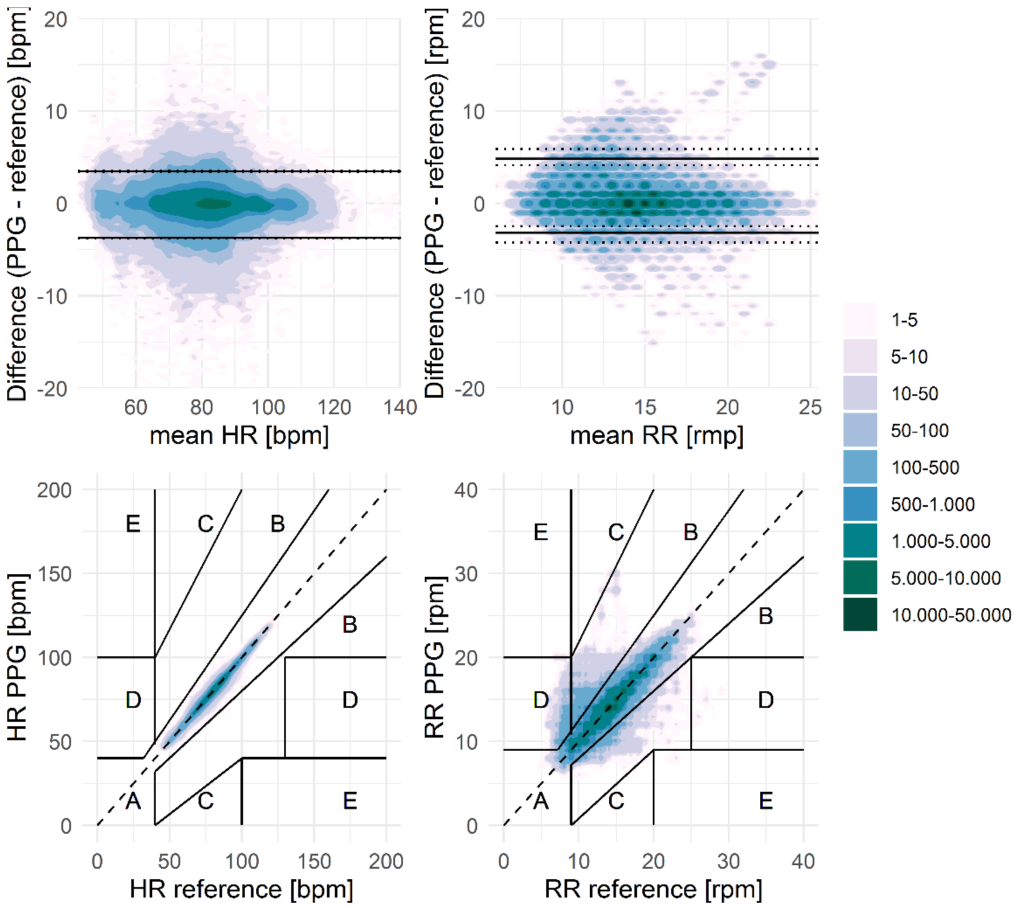

Supplemental figure 1. Bland–Altman (top) and Clarke error grid (bottom) plots of the vital parameters obtained from the PPG wristband and reference monitor in patients admitted to the ICU, each data point represents one second.

Supplemental table 1. Agreement and clinical accuracy of heart rate (HR) and respiratory rate (RR) measured by the PPG wristband compared to the reference monitor in patients admitted to the ICU.

	HR	RR
Recording length	2.60 h [26.5 min – 10.2 h]	4.42 h [26.6 min – 10.9 h]
% Good quality PPG	96 [92-98]	33 [11-52]
Gap length	8 [3-16]	19 [9-40]
% gaps < 60 sec	96%	83%
Pearsons correlation coef	0.99	0.83
Bias (sd)	-0.14 (1.8)	0.83 (2)
Lower limmit of agreement	-3.76 [-3.8: -3.7]	-3.18 [-4.2 : -2.5]
Upper limit of agreement	3.47 [3.4 : 3.5]	4.83 [4.1 : 5.9]
% within 5bpm or 3 rpm	97	95

Post-Anaesthesia Care Unit

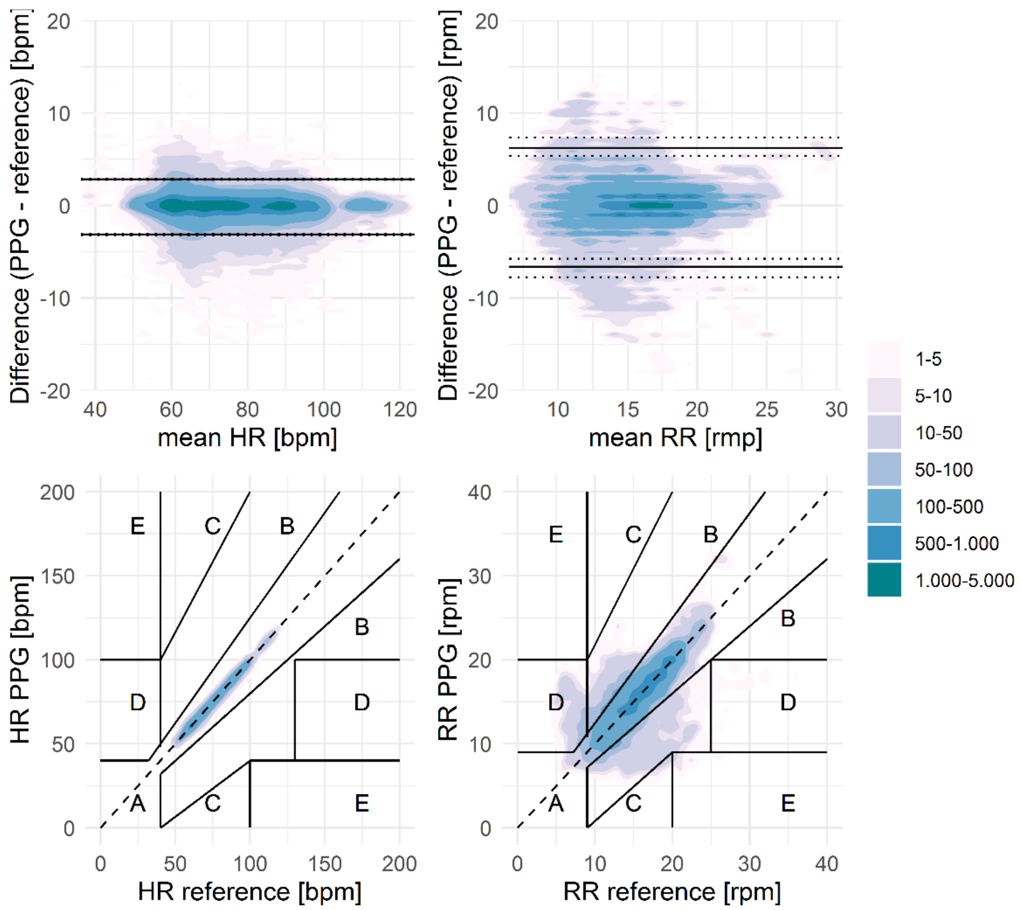

Supplemental figure 2. Bland–Altman (top) and Clarke error grid (bottom) plots of the vital parameters obtained from the PPG wristband and reference monitor in patients admitted to the PACU, each data point represents one second.

Supplemental table 2. Agreement and clinical accuracy of heart rate (HR) and respiratory rate (RR) measured by the PPG wristband compared to the reference monitor in patients admitted to the PACU.

	HR	RR
Recording length	0.83 h [15.9 min – 5.93 h]	0.88 h [15.9 min - 5.9 h]
% Good quality PPG	98 [93-99]	16 [6-32]
Gap length	7 [4 -15]	27 [12-64]
% gaps < 60 sec	95 %	73 %
Pearsons correlation coef	0.99	0.7
Bias (sd)	-0.16 (1.5)	-0.2 (3.3)
Lower limmit of agreement	-3.14 [-3.2 : -3.1]	-6.62 [-7.8 : -5.8]
Upper limit of agreement	2.83 [2.8 : 2.9]	6.21 [5.4 : 7.4]
% within 5bpm or 3 rpm	98	88
